# Using Plantain Rachis Fibers and Mopa-Mopa Resin to Develop a Fully Biobased Composite Material

**DOI:** 10.3390/polym16030329

**Published:** 2024-01-25

**Authors:** Valeria Sánchez Morales, Brenda Alejandra Martínez Salinas, Jose Herminsul Mina Hernandez, Estivinson Córdoba Urrutia, Lety del Pilar Fajardo Cabrera de Lima, Harry Maturana Peña, Alex Valadez González, Carlos R. Ríos-Soberanis, Emilio Pérez-Pacheco

**Affiliations:** 1 Escuela de Ingeniería de Materiales, Grupo Materiales Compuestos, Universidad del Valle, Calle 13 No. 100-00, Cali 76001, Colombia; valeria.sanchez@correounivalle.edu.co (V.S.M.); brenda.martinez@correounivalle.edu.co (B.A.M.S.); 2 Grupo de Investigación en Ciencia Animal y Recursos Agroforestales, Universidad Tecnológica del Chocó, Carrera 22 No. 18B-10B, Quibdó 270001, Colombia; estivinson.cordoba@utch.edu.co; 3 Grupo Tribología, Polímeros, Metalurgia de Polvos y Transformaciones de Residuos Sólidos, Universidad del Valle, Calle 13 No. 100-00, Cali 76001, Colombia; letydelpilar.fajardo@correounivalle.edu.co; 4 Grupo de Investigación en Desarrollo de Materiales y Productos—GIDEMP, Centro Nacional de Asistencia Técnica a la Industria (ASTIN), SENA, Calle 52 No 2bis 15, Cali 760003, Colombia; harry.mp@misena.edu.co; 5 Unidad de Materiales, Centro de Investigación Científica de Yucatán, A.C., Calle 43 #. No. 130, Col. Chuburná de Hidalgo, Mérida C.P. 97205, Yucatán, Mexico; avaladez@cicy.mx (A.V.G.); rolando@cicy.mx (C.R.R.-S.); 6 Tecnológico Nacional de México, Campus Instituto Tecnológico Superior de Calkiní, Cuerpo Académico Bioprocesos, Av. Ah-Canul, Calkiní C.P. 24900, Campeche, Mexico; eperez@itescam.edu.mx

**Keywords:** plantain waste, Mopa-Mopa resin, biobased composite material, biocomposite materials, plantain fibers, barniz de pasto

## Abstract

A completely biobased composite material was developed using a matrix of natural resin extracted from the *Elaegia pastoensis* Mora plant, commonly known as Mopa-Mopa or “Barniz de Pasto”, reinforced with fibers extracted from plantain rachis agricultural residues. A solvent process, involving grinding, distillation, filtration, and drying stages, was implemented to extract the resin from the plant bud. To obtain the resin from the plant bud, the vegetable material was ground and then dissolved in a water-alcohol blend, followed by distillation, filtration, and grinding until the powdered resin was ready for use in the preparation of the biocomposite. Likewise, using mechanical techniques, the plantain rachis fibers were extracted and worked in their native condition and with a previous alkalinization surface treatment. Finally, the biocomposite material was developed with and without incorporating stearic acid, which was included to reduce the material’s moisture absorption. Ultimately stearic acid was used as an additive to reduce biocomposite moisture absorption. The tensile mechanical results showed that the Mopa-Mopa resin reached a maximum strength of 20 MPa, which decreased with the incorporation of the additive to 12 MPa, indicating its plasticization effect. Likewise, slight decreases in moisture absorption were also evidenced with the incorporation of stearic acid. With the inclusion of rachis plantain fibers in their native state, a reduction in the tensile mechanical properties was found, proportional to the amount added. On the other hand, with the alkalinization treatment of the fibers, the behavior was the opposite, evidencing increases in tensile strength, indicating that the fiber modification improved the interfacial adhesion with the Mopa-Mopa matrix. On the other hand, the evaluation of the moisture absorption of the biocomposite material evidenced, as expected, that the absorption level was favored by the relative humidity used in the conditioning (47, 77, and 97%), which also had an impact on the decrease of the mechanical tensile properties, being this was slightly counteracted by the inclusion of stearic acid in the formulation of the material.

## 1. Introduction

In the jungle regions of Colombia, there are numerous promising species of various types. One example is the vegetable resin commonly known as Mopa-Mopa (MM), which comes from the fruit and bud of the *Elaeagia pastoensis* Mora plant. This wild shrub is found in forested areas of the Andean-Amazonian piedmont of the Departments of Putumayo, Cauca, Caquetá, and Nariño, as well as in nearby dispersion zones, at altitudes ranging from 1400 to 2000 m above sea level, in a humid tropical climate [1,2,3,4]. Although Mopa-Mopa possesses some physicochemical characteristics that make it attractive for consideration in the field of biocomposite materials, over the years, it has been extracted and benefited by generations of farmers to be used as raw material for the decoration of handicrafts, mainly [5,6]. In this sense, the literature shows few scientific studies [7,8,9,10] that report evaluations related to the use of this resin, which opens a wide field to investigate its characteristics and take better advantage of it in the scientific area [9]. After its extraction process, Mopa-Mopa resin is characterized by an emerald green color, no odor or taste, and a semi-solid consistency that requires prior drying to be pulverized. It also has a density of 1.108 g/cm^3^, a semi-crystalline structure (40% relative crystallinity) with a temperature and enthalpy of fusion of 146 °C and 104 J/g, respectively. The material is also thermally stable up to 230 °C, which makes it feasible for thermal processing [8,9,10].

On the other hand, throughout history, natural fibers of plant origin, known as lignocellulosic materials, found in nature mostly as agricultural and agro-industrial waste, have been promoted and increased [11]. Such is the case of plantain rachis, located in the fraction of the plant that supports the fruit set and is constituted by semi-woody fibers, a waste that, when decomposing, hurts the environment due to the growth of different microorganisms [12]. According to production and market indicators of the plantain chain in Colombia, plantain is the most widely planted crop and the most important regarding food security; by 2020, plantain production was approximately 4.5 million tons in more than 500 thousand hectares grown [13]. This makes the rachis a potential resource due to its great availability, with fibers that can provide good mechanical properties to polymeric matrices, giving them an added value to produce biocomposite materials on a massive scale in a circular economy. Plantain fibers consist of 71.08% cellulose, 12.61% hemicellulose, and 7.67% lignin in their chemical composition, likewise a density of 1.28 g/cm^3^, microfibrillar angle of 11°, a diameter of 138 µm, a tensile strength of 412.5 MPa, and a strain at break of 27.89% [14].

Due to the negative environmental impact generated by conventional synthetic polymers, when they are not properly disposed of at the end of their useful life, multiple types of research are currently being carried out focused on the development of compostable biobased materials, which come from natural biomasses such as proteins, starches, hydroxyalkanoates, and chitin among others [15,16,17,18,19,20,21]. Currently, the use of materials of natural origin is being chosen, which benefits society by favoring the non-destruction of the environment [22,23]. This would be the case with the completely biobased composite material of the Mopa-Mopa resin matrix (continuous phase) and plantain rachis fiber reinforcement (discontinuous phase) developed in the present work. Here, we started with the implementation of extraction processes to obtain the resin and plantain fibers, as well as the subsequent transformation to obtain the biocomposite material in which we additionally studied the influence of the incorporation of stearic acid (lubricant) to reduce the percentage of moisture absorption and the surface treatment of the plantain fibers seeking to improve the interfacial adhesion with the matrix. Finally, it is expected that this type of development contribute to a circular economy by the use of agricultural residues that can be converted into resources that can be reintroduced into the productive apparatus, visualizing possible applications, especially in decorative furniture for interiors, where conventional materials based on wood and synthetic polymers can begin to be replaced, in a trend that currently points towards the manufacture of what has been called sustainable furniture, among other applications [24].

## 2. Materials

**Figure 1:**
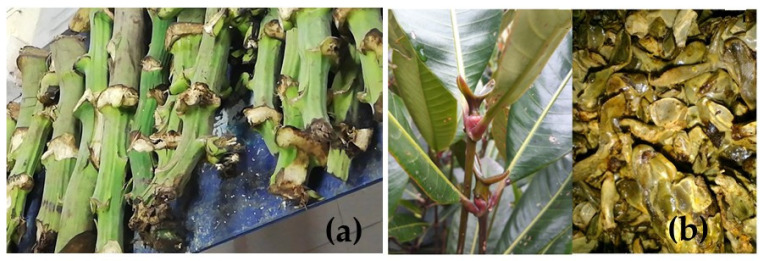
Raw materials for producing biobased composite material: (**a**) plantain rachis agricultural residues; (**b**) *Elaegia pastoensis* Mora plant and seed.

The starting point was the rachis or stems (Figure 1a), agricultural residues located in the Alameda marketplace in Santiago de Cali to obtain the plantain fibers. From this rachis, long fibers were extracted mechanically and later arranged as discontinuous reinforcement in a matrix based on Mopa-Mopa resin. The buds for obtaining Mopa-Mopa resin (Figure 1b) come from the *Elaegia pastoensis* Mora plant, native to the Department of Putumayo; the taxonomic classification of the plant has been described in previous works [10]. To carry out the extraction process of the Mopa-Mopa resin, 96% pure ethanol purchased from Genquímicos (Santiago de Cali, Colombia) was used, and sodium hydroxide used in the alkaline treatment of the fibers was reactive grade acquired by the company Técnica Química S.A. (Santiago de Cali, Colombia). Finally, for the extraction of the Mopa-Mopa resin, distilled water obtained from the Polymeric Materials Laboratory of the Universidad del Valle was used, using a BOE8704200 model BOECO water distiller (Rödingsmarkt, Hamburg, Germany). This technique was used to purify tap water, obtaining water with a high degree of purity.

## 3. Experimental Procedure

### 3.1. Obtaining the Mopa-Mopa Resin

**Figure 2:**
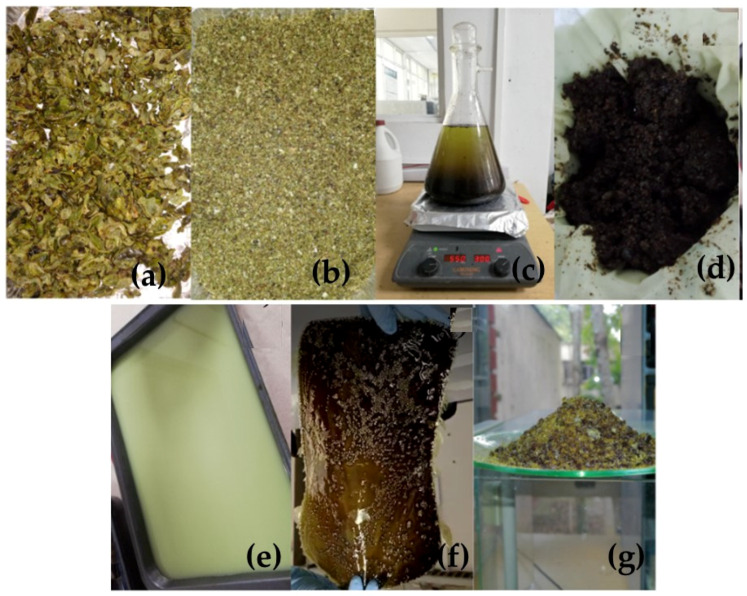
Resin extraction process, (**a**) Mopa-Mopa bud in vegetable state; (**b**) Ground Mopa-Mopa bud; (**c**) Distillation process of Mopa-Mopa and ethanol; (**d**) Residue from filtration process; (**e**) Precipitation of Mopa-Mopa resin; (**f**) Extracted Mopa-Mopa resin; (**g**) Mopa-Mopa resin after grinding.

For the extraction of the Mopa-Mopa resin, the buds of the *Elaegia pastoensis* Mora plant were cooled at a temperature of 5 °C to facilitate the subsequent grinding process in the Fritsch cutting mill pulverisette 15 (Idar-Oberstein, Rheinland-Pfalz, Germany), with which the size of the buds was reduced until particles were obtained, thus increasing its surface area, to facilitate the obtaining of the resin. The ground buds were added to a 3 L Kitasato flask containing a solution of ethanol in distilled water at a concentration of 20% (*m*/*v*), and a distillation system was installed where the solution was kept at a temperature of 75 °C for 25 min. The resin was then filtered using a porcelain funnel and an absorbent towel, which allowed the separation of solid and organic residues and achieved greater purity. Likewise, 1500 mL of distilled water was heated to 100 °C in another container and added to the concentrated solution of Mopa-Mopa, previously filtered and maintaining constant agitation. Next, resin precipitation was promoted with the consequent liquid phase separation as part of the solvent from the previous step evaporated. The precipitated resin was deposited in a tray and cooled for 15 min to room temperature. Subsequently, the trays were placed in a Binder FD-115 oven (Tuttlingen, Baden-Württemberg, Germany) at 60 °C for 24 h. After this time, the precipitated resin was dried in the oven. After this time, the resin was removed from the oven and cooled to 5 °C to finally be ground in a Metvisa LQ-8 industrial blender (Li-moeiro, Pernambuco, Brazil) to reduce its size to a sieve number 4 (4.75 mm). Figure 2 shows a schematic representation of the extraction process followed.

### 3.2. Extraction of Plantain Rachis Fibers

**Figure 3:**
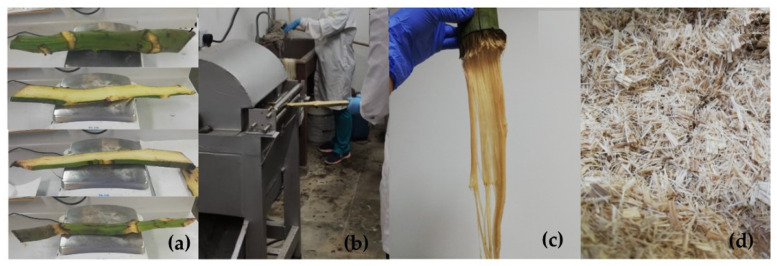
Extraction of plantain fibers: (**a**) cut rachis sheets; (**b**) rachis shredding process; (**c**) obtained fibers; (**d**) cut fibers after drying.

To extract the plantain fibers, longitudinal cuts were previously made to the rachis to obtain sheets (see Figure 3a) that were repeatedly subjected to mechanical blows in equipment with rotating blades (see Figure 3b) to almost eliminate the binding material from the fiber. Subsequently, the fibers obtained (Figure 3c) were washed with water to eliminate the residual binder. Then, with the help of a comb, the fibers were separated and dried at 60 °C for 24 h in a Binder FD-115 oven, achieving a moisture content between 5% and 10%.

Finally, the fibers were cut to lengths of approximately 5 mm (see Figure 3d). This whole extraction process can be seen schematically in Figure 3, which shows the step-by-step process from cutting the sheets to obtaining the fibers used in the biocomposites.

### 3.3. Alkalinization Process of Plantain Fibers

The lignocellulosic fibers extracted from plantain rachis were modified superficially through an alkalinization treatment. In this sense, the fibers, previously obtained, were immersed in an aqueous solution of sodium hydroxide (NaOH) at 2% *m*/*v* for 1 h at room temperature, then washed with distilled water until reaching a neutral pH, and finally, the fibers were dried in an oven at 60 °C for 12 h to be cut to a length of approximately 5 mm. These conditions were similar to those followed in some investigations to superficially modify other natural fibers, such as fique [10] and henequen [25], similar to those extracted from plantain.

### 3.4. Preparation of Biobased Composite Material

**Figure 4:**
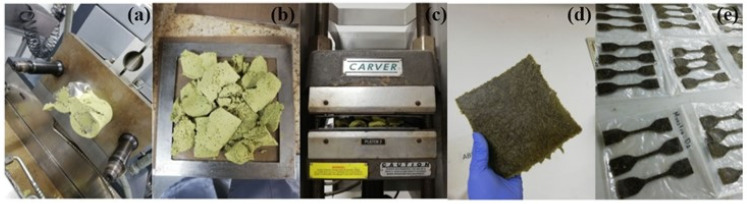
Composite preparation process: (**a**) material coming out of the blender; (**b**) material before compression molding; (**c**) hydraulic press with the blend inside; (**d**) plate obtained; (**e**) die-cut specimens.

The biobased composite material was processed by mixing the Mopa-Mopa resin with the three different percentages of native and alkalized fibers (5%, 10%, and 15%), maintaining a constant length of 5 mm, using a Thermo Scientific internal torque mixer model Haake Rheomix OS (Waltham, MA, USA) as shown in Figure 4a, at a temperature of 130 °C and a spindle speed of 50 rpm for 5 min, for each of the formulations of the experimental design specified in Table 1. Likewise, the effect of the additional incorporation of an additive (1% of stearic acid) in some of these formulations was also considered. The composite was then processed by hot compression molding using a semi-automatic hydraulic press equipped with heating plates and cooling by forced water circulation at a temperature of 130 °C and a pressure of 100 KN/m^2^ for 8 min, obtaining laminates (Figure 4b–d) that were passed through an IAC 6090 CNC Router Machine (Santiago de Cali, Valle del Cauca, Colombia) to obtain ASTM D638 standard type IV test specimens [26]. The experimental design that served as a basis to study the effect of the incorporation of fibers to the Mopa-Mopa resin matrix was adjusted in two blocks, with fiber percentages of 5%, 10%, and 15%, both for native fibers (without additive) and alkalized fibers (with additive), as can be observed in Table 1.

### 3.5. Fourier Transform Infrared Spectroscopy (FT-IR)

For the compositional analysis of the Mopa-Mopa resin, a Perkin Elmer Spectrum 100 FT-IR Spectrometer (Bridgeport, CT, USA) with attenuated total reflectance (ATR) accessory (ZnSe crystals) was used. All these analyses were performed at 100 scans and a resolution of 4 cm^−1^ in the spectral range 4000–600 cm^−1^.

### 3.6. Moisture Absorption

The moisture absorption test of the Mopa-Mopa resin and the biobased composite materials was carried out by conditioning the materials to three relative humidities corresponding to 47%, 77%, and 97% from the use of glass desiccators provided with potassium carbonate, sodium chloride, and potassium sulfate salts, respectively; according to the protocol described in the ASTM E104 standard [27]. The test specimens were previously subjected to oven drying at 80 °C for 12 h, taking their initial mass; after they were incorporated into the desiccators, a continuous record of the mass variation with time was made for 30 days. The moisture absorption was determined with the help of the model presented in Equation (1).

(1) $$MA=\left(\frac{Mt-Ms}{Ms}\right)\times 100$$

where *Ms* is the initial mass; *Mt* is the mass variation with time, and the *MA* is the moisture absorption.

### 3.7. Scanning Electron Microscopy (SEM)

The morphological characterization was carried out on the cross-section of the Mopa-Mopa resin and the biobased composite material after a stress test was performed. A JEOL scanning electron microscope model JSM 6490L (Jeol, Mexico D.F., Mexico), operated at a voltage of 25 kV in high vacuum mode, with secondary electrons at 500× magnification, was used. The samples were previously coated with a layer of gold using a Denton Vacuum Desk IV cold spray coater model STANDARD A PHENOM (Moorestown, NJ, USA).

### 3.8. Tensile Test

The tensile mechanical properties were determined for the Mopa-Mopa resin and the biobased composite material in a Tinius Olsen model H50KS (Horsham, PA, USA) universal testing machine according to the ASTM D638 standard. The Mopa-Mopa resin and the biobased composite material, type IV specimens were tested at a head displacement rate of 5 mm/min. Two kinds of specimens were tested: one at time t1 (zero days), i.e., immediately after a drying process of the specimens for 12 h in the Binder FD-115 oven at a temperature of 80 °C; and the second at a time t2 (30 days) after conditioning the specimens at different relative humidities.

## 4. Results and Discussion

### 4.1. Fourier Transform Infrared Spectroscopy (FT-IR)

**Figure 5:**
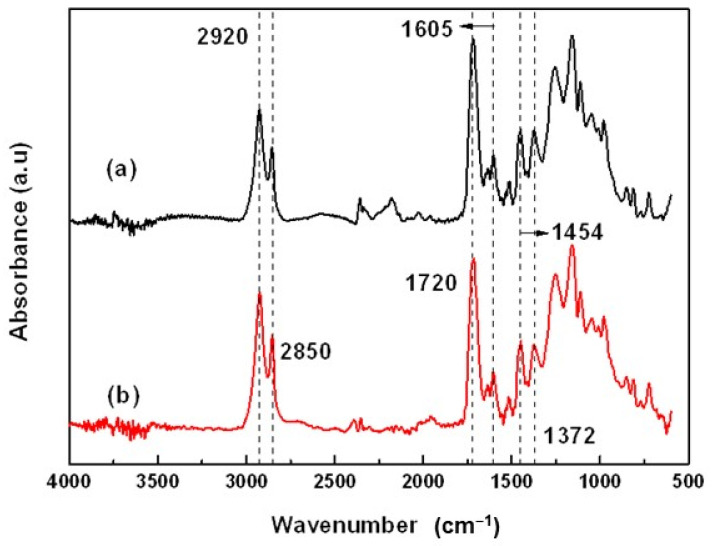
FT-IR of (**a**) Mopa-Mopa resin without additive; (**b**) Mopa-Mopa resin with additive.

The infrared spectra of the Mopa-Mopa resin specimens without and with additive (stearic acid) are shown in Figure 5. These specimens were evaluated at time zero, that is, immediately after they were manufactured by hot compression molding. In the spectrum of Figure 5a, it can observe the presence of absorption peaks that correspond to the symmetric and asymmetric stretching of the methylene group (-CH_2_-) at 2920 and 2850 cm^−1^, respectively, the tension stretching of the carbonyl group -C=O at 1720 cm^−1^ and the stretching of the aromatic C=C bond at 1605 cm^−1^. Likewise, absorption bands can be observed due to the bending of -CH_2_- and the methyl group (-CH_3_) at 1454 and 1372 cm^−1^, respectively. Between 1300 and 1000 cm^−1^, materials of biological origin usually present signals that are not very clear since the material is formed from different metabolites with functional groups that present peaks in this region of the spectrum. These absorption bands have been reported as characteristics of the Mopa-Mopa resin since it is made up, among others, of phenylpropanoids and lipophilic flavonoids [28]. Insuasty et al. [7] and Mina et al. [8] reported similar findings in studies on Mopa-Mopa resin.

Regarding the resin containing stearic acid as an additive (Figure 5b), it can be seen that its infrared spectrum is practically similar to that of the resin without an additive. This is to be expected because the main absorption peaks of the additive at 2912, 2845, 1720, and 1460 cm^−1^ overlap with those of the Mopa-Mopa resin. In addition to this, the concentration of the additive used was 1%, which makes its detection difficult using FTIR.

### 4.2. Scanning Electron Microscopy (SEM)

**Figure 6:**
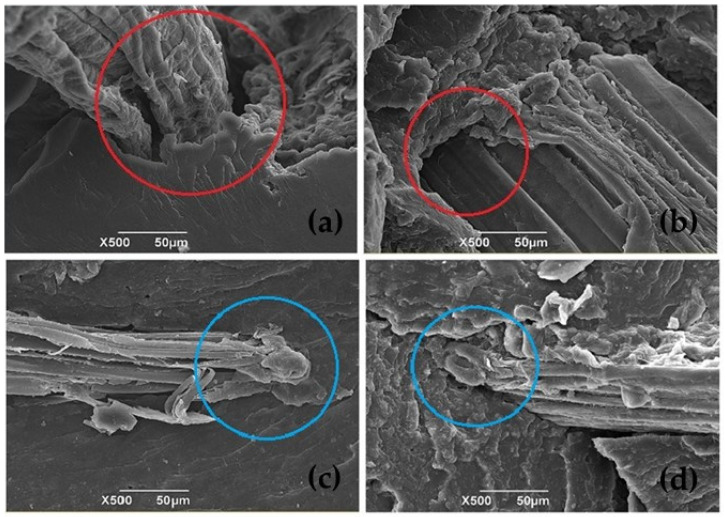
SEM micrographs of (**a**) Mopa-Mopa with native fiber NF/5; (**b**) Mopa-Mopa with native fiber with additive NF/A/5; (**c**) Mopa-Mopa with alkalized fiber AF/15; (**d**) Mopa-Mopa with alkalized fiber with additive AF/A/15. The red and blue circles relate to the quality of the interfacial zone.

A scanning electron microscopy (SEM) analysis was performed on the biobased composite material to compare its morphology, especially to visualize the area between the fibers and the matrix, seeking to establish the possible influence of the alkalinization surface treatment on the bonding of the fibers. The SEM images in Figure 6a,b show the cross-sectional tensile-fracture surface corresponding to the reinforcement with the native plantain fibers, with and without additive, respectively. Here, it can be observed that the sample without additive showed poor adhesion (see red circles) compared with the additivated material, for which the adhesion was slightly improved. This behavior was probably because, due to its lubricant nature, the incorporation of the additive resulted in a greater fluidity of the matrix during the processing of the biocomposite material, reducing the generation of defects and promoting a more significant presence of the Mopa-Mopa resin in the interfacial zone with the plantain fibers.

On the other hand, the micrographs in Figure 6c,d show the images of the biocomposite reinforced with the alkalized fibers, with and without additives, respectively. Comparing these samples with those of the untreated fibers, it can be seen that the alkalinization pretreatment promoted a better adhesion in the fibers that was reflected in a more coherent interfacial zone (see blue circle), being the best case for the material with additive in the matrix and treated fiber, which incidentally was the one that presented the best behavior in the tensile mechanical tests.

This behavior has been reported for natural fique, jute, and coconut fibers. It has been attributed to an increase in the mechanical component of adhesion (mechanical interlocking) because the alkaline treatment increases the roughness of the fibers [29,30,31,32].

### 4.3. Tensile Test

Table 2 shows the tensile mechanical properties calculated as the average value of three measurements associated with each formulation proposed in the experimental design specified in Table 1. In the first instance, these tests were carried out at zero time, immediately after the hot compression molding of the biocomposite material to obtain the test samples; here, native and alkalized fiber contents of 5%, 10%, and 15% by weight were used, in addition to the incorporation of stearic acid as an additive to reduce the degree of moisture absorption of the material. The data in Table 2 shows that the inclusion of plantain rachis fibers diminishes the mechanical properties of Mopa-Mopa resin without reinforcement, as shown in Table 3. Moreover, it’s evident the decrease depends on the number of fibers incorporated. In particular, the NF/5 and NF/A/5 samples, related to the incorporation of native fibers at 5%, exhibited better mechanical behavior with tensile mechanical strength values of 16.80 and 15.89 MPa, respectively. On the other hand, in the case of samples AF/15 and AF/A/15, associated with the addition of fibers with alkalinization treatment, the formulations with the best tensile performance were those containing a higher percentage by weight of fibers, precisely 15%, resulting in values of 14.80 and 18.19 MPa, respectively. The above shows the low compatibility of the native fiber with the Mopa-Mopa matrix, which turns it into a defect, not only with a null reinforcement but also to the detriment of the mechanical behavior. The above was previously evidenced in the SEM micrographs shown in Figure 6, where it was possible to appreciate the generation of poor interfacial zones in the case of untreated fibers, which improve when the process of alkalinization of the fibers is carried out. This phenomenon has been reported in other systems where the fibers and the matrix have low compatibility [31].

**Figure 7:**
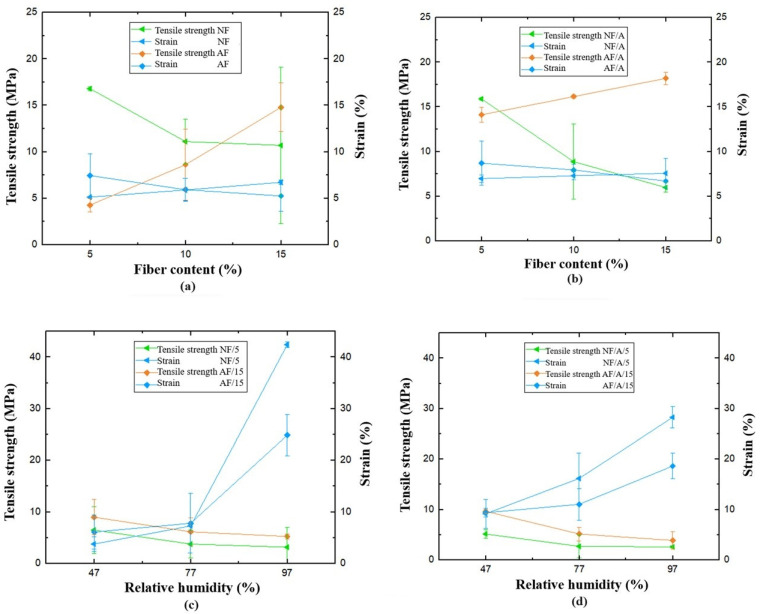
Maximum strength and strain values for biobased composite material worked with (**a**) NF and AF at 5, 10, and 15% of fibers without additive; (**b**) NF/A and AF/A at 5, 10, and 15% of fibers with additive; (**c**) NF/5 and NF/A/5 at 47%, 77%, and 97% R.H.; (**d**) AF/15 and AF/A/15 at 47%, 77%, and 97% R.H.

In the second part of this characterization and based on the data obtained, it was established that the samples of the biocomposite material with the best mechanical behavior would be evaluated in tension after conditioning for 30 days at different relative humidities (47%, 77%, and 97%). In this case, the best-performing formulations corresponded to the composite with 5% native fibers (NF/5) and to the material reinforced with 15% alkalized fibers (AF/A/15). Figure 7 shows the maximum tensile strength and deformation as a function of the relative humidities of interest. As expected, it was found that the mechanical behavior of the material turned out to be an inverse function of the relative humidity used in the conditioning. In this sense, increasing the relative humidity decreased the maximum tensile strength and increased the maximum deformation, indicating that the absorbed water molecules worked as a plasticizer in the material. In the case of the formulations with 5% native fibers, it was evidenced that the addition of stearic acid promoted the decrease of its maximum tensile strength, from values of 16.8 to 15.9 MPa for the mixtures without and with additives, respectively. Likewise, when the material was subjected to the highest relative humidities, it decreased up to 80% of its resistance. In comparison, in the case of the composite with 15% alkalized fibers, the decrease was 60%. This phenomenon is characteristic of materials with a hydrophilic nature and has been reported in materials such as thermoplastic starch composites reinforced with natural fibers [32]. Similarly, it is essential to note that the chemical composition of natural fibers directly influences their moisture adsorption level [33], which also conditions the mechanical properties of the biocomposite material, probably by promoting a weakening of the interfacial zone with the matrix [34,35,36,37].

### 4.4. Moisture Absorption

**Figure 8:**
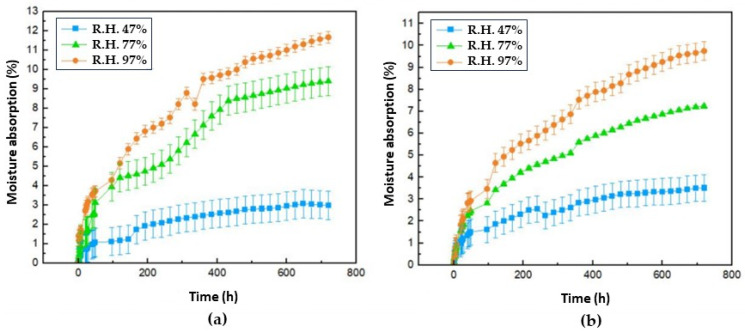
Moisture absorption isotherms at 47%, 77%, and 97% R.H. with different conditioning times for the samples (**a**) Mopa-Mopa resin without additive; (**b**) Mopa-Mopa resin with additive.

Figure 8 shows the moisture absorption isotherms corresponding to samples Figure 8a (Mopa-Mopa resin without additive) and Figure 8b (Mopa-Mopa resin with additive), where it is evident for both cases that the increase in mass due to moisture absorption is a direct function of the relative humidity used for the conditioning of the material, thus affecting the mechanical properties as the water molecules act as a plasticizing agent in the structure of the material, as previously mentioned when studying the tensile mechanical properties of the biocomposite material. The lowest equilibrium absorption values corresponded to the samples conditioned at 47% relative humidity, followed by those at 77% and 97%, respectively. This behavior followed the same trend regardless of whether the lubricant additive was included in the formulation of the material. However, it was evident that with the latter, the absorption became slightly lower, which was a phenomenon that was expected due to the hydrophobic nature of the stearic acid used [38,39,40,41,42]. These values indicated that the Mopa-Mopa resin obtained presented a high percentage of moisture absorption (11.68 and 9.75, without and with additive) in comparison with the results reported by Mina et al. [10], who found values in the order of 2.5% for Mopa-Mopa resin conditioned at 97% relative humidity, probably due to the variation of this biobased material that is a function of the botanic source, in addition to the different levels of purity that can be achieved in the extraction. On the other hand, if another type of biobased resin is taken as a reference, such as thermoplastic starch, which reaches absorption values of 7 [43] and 60% [44] for relative humidities of 54 and 97%, respectively, the Mopa-Mopa resin presents a better performance.

**Figure 9:**
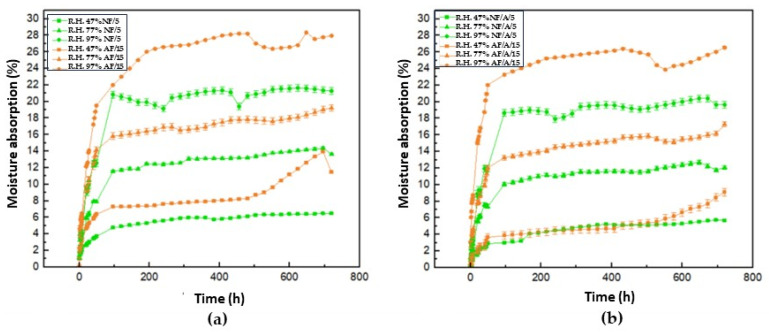
Moisture absorption isotherms at 47%, 77%, and 97% R.H. with different conditioning times for the samples: (**a**) NF/5 and AF/15; (**b**) NF/A/5 and AF/A/15.

Since Mopa-Mopa resin has proven to be a hydrophilic material, as evidenced in previous characterizations, and plantain fibers also possess the capacity to adsorb moisture, it can be observed in Figure 9 that the absorption isotherms corresponding to the biocomposite materials with 5% native fiber (Figure 9a) and 15% alkalized fiber (Figure 9b) show an increase in the absorption percentage that is a direct function of the value of the relative humidity under which the conditioning was carried out. The values achieved in this research were higher than the 12% moisture absorption reported by Mina et al. [10] for Mopa-Mopa resin composites and alkalized fique fibers (Table 4), studied at 97% humidity, who also evidenced the relationship between the increase in fiber content and mass gain in the material. Additionally, in these absorption isotherms, the positive effect of incorporating the additive in decreasing the moisture absorption value of the composite materials can be observed, like what happened previously with the Mopa-Mopa resin without reinforcement. When native fibers were used in the Mopa-Mopa matrix, the absorption percentage achieved was 5%, while when the fibers were incorporated with the alkaline treatment, values of 15% were reached. Here, the increase in moisture absorption is influenced by the amount of fibers present in the material. Specifically, the composite with alkalized fibers, which has a higher percentage of reinforcement, presents higher values compared to the composite containing native fiber, which is present in lower quantities. This is because a higher number of fibers during the forming process increases the probability of creating pores or micropores in the matrix-reinforcement interface, which facilitates the penetration of water molecules into the material. In addition, as the fibers had a surface treatment of alkalinization, this also favors moisture absorption due to a relative increase in cellulose concentration at the surface level.

## 5. Conclusions

It was possible to obtain a fully biobased composite material by using a natural resin (Mopa-Mopa) extracted from the buds of the *Elaegia pastoensis* Mora following conventional experimental processes implemented in the research (distillation, grinding, and drying), reinforced with fibers from agricultural waste (plantain rachis). This biocomposite material could be processed using conventional transformation processes such as hot compression molding and reached mechanical tensile strength close to 20 MPa, which is a significant value considering that it is a material that does not contain synthetic components and is partially based on waste.

The incorporation of stearic acid in the biocomposite material promoted a decrease in the level of moisture absorption of the material, which was maintained at the different relative humidities used in the conditioning. This positive effect could be evaluated at proportions higher than the 1% used in this research, aiming to achieve lower losses in the mechanical behavior due to the plasticizing effect generated by the water molecules absorbed by the material.

The alkali-surface treatment applied to the plantain rachis fibers improved the interfacial zone with the Mopa-Mopa resin-based matrix, which was evidenced by a greater adhesion through SEM micrographs of the rupture surface in samples previously fractured in tension. Additionally, unlike the work with native fibers, in this case, the mechanical tensile strength was a direct function of the fiber content for the three percentages worked in the experiment, suggesting that the performance of the biocomposite material can be further improved with better surface treatment and an increase in the percentage of reinforcement. Finally, due to the properties obtained, the biobased composite material developed is susceptible to replacing some conventional materials (wood, plastic) in the manufacture of non-structural elements, such as rigid packaging or panels for interior use. Among other non-structural architectural elements of interior design (decorative furniture and educational furniture).

## Figures and Tables

**Table 1:** Experimental design for the formulations of biobased composite material worked.

	Factors	Level 1 (with Additive) Sublevels			Level 2 (without Additive) Sublevels		
Block 1	Fiber content Test specimen *	5% NF/A/5	10% NF/A/10	15% NF/A/15	5% NF/5	10% NF/10	15% NF/15
	Fiber type	Native	Native	Native	Native	Native	Native
Block 2	Fiber content Test specimen **	5% AF/A/5	10% AF/A/10	15% AF/A/15	5% AF/5	10% AF/10	15% AF/15
	Fiber type	Alkalized	Alkalized	Alkalized	Alkalized	Alkalized	Alkalized

* NF: Native fiber; A: Additive; 5, 10, and 15: Content fiber. ** AF: Alkalized fiber; A: Additive; 5, 10, and 15: Content fiber.

**Table 2:** Maximum strength and strain at break values for the formulations of biobased composite studied.

Block	Level	Test Specimen	Additive Content	Fiber Type	Fiber Content (%)	Tensile Strength (MPa)	Strain (%)
1	1	NF/A/5	with	Native	5	15.9	7.0
		NF/A/10			10	8.9	7.3
		NF/A/15			15	6.0	7.6
	2	NF/5	without	Native	5	16.8	5.1
		NF/10			10	11.1	5.9
		NF/15			15	10.7	6.7
2	1	AF/A/5	with	Alkalized	5	14.1	8.7
		AF/A/10			10	16.2	8.0
		AF/A/15			15	18.2	6.7
	2	AF/5	without	Alkalized	5	4.3	7.5
		AF/10			10	8.6	5.9
		AF/15			15	14.8	5.3

**Table 3:** Maximum strength and strain values were achieved for samples NF/5 and AF/15 of the composite material without additive and samples NF/A/5 and AF/A/15 of the composite material with additive of the best-performing blends.

Test Specimen	Additive Content	Time (D)	Relativity Humidity (%)	Tensile Strength (MPa)	Strain at Break (%)
Mopa-Mopa	with	0	-	11.8	7.1
		30	47	8.9	31.4
			77	8.1	79.9
			97	4.5	80.9
	without	0	-	20.9	9.0
		30	47	18.1	13.0
			77	12.1	25.8
			97	6.3	26.1
NF/A/5	with	0	-	15.9	7.0
		30	47	5.2	9.2
			77	2.7	16.2
			97	2.6	28.3
NF/5	without	0	-	16.8	5.1
		30	47	6.5	3.8
			77	3.8	7.3
			97	3.2	42.4
AF/A/15	with	0	-	18.2	6.7
		30	47	9.7	9.4
			77-	5.2	11.1
			97	3.9	18.7
AF/15	without	0	-	14.8	5.3
		30	47	9.0	6.1
			77	6.2	7.8
			97	5.3	24.9

**Table 4:** Moisture absorption contents for Mopa-Mopa resin and biobased composite materials from experimental data and literature reports.

Matrix Type	Fiber Type	Fiber Content (%)	Moisture Absorption (%)				Reference
			47	77	97	Other Moisture	
Mopa-Mopa	N/A	0	0.09	0.64	1.90	-	[10]
	Fique untreated	10	0.54	2.20	4.96	-	
		20	0.46	2.04	6.63	-	
	Fique alkalized	10	0.77	3.34	7.00	-	
		20	0.88	3.95	12.32	-	
Mopa-Mopa with additive	N/A	N/A	2.97	9.41	11.68	-	Experimental date
	Plantain untreated	5	5.61	12.07	19.65	-	
	Plantain alkalized	15	9.11	17.27	26.54	-	
Mopa-Mopa without additive	N/A	0	3.51	7.23	9.75		
	Plantain untreated	5	6.47	13.65	21.28	-	
	Plantain alkalized	15	11.46	19.22	27.96		
Corn TPS	-	-	-	-	60.00	11.4 (53%)	[44]
Cassava TPS	-	-	-	-	-	7.00 (54%)	[45]
						1.00 (29%)	
Corn TPS	-	-	-	-	-	9.30 (53%)	[46]
	Agave bagasse	10	-	-	-	8.64 (53%)	
		15	-	-	-	8.52 (53%)	

## Data Availability

Data are contained within the article.
